# Osteopathic manipulative treatment for pneumonia

**DOI:** 10.1186/1750-4732-4-3

**Published:** 2010-03-19

**Authors:** Murray Goldstein

**Affiliations:** 1 Cerebral Palsy International Research Foundation, Washington, DC, USA

## Abstract

The pneumonias due to infection continue to be a meaningful threat to the health and viability of persons, particularly those in high risk groups: children, the aged and the debilitated. Noll and colleagues provide us with the results of a well-designed and well-executed multi-institutional controlled clinical trial to evaluate the efficacy of osteopathic manipulative treatment (OMT) in the treatment of pneumonia. The data obtained indicate that by intention-to-treat analysis, the addition of OMT to conventional care did not improve the designated outcomes when compared to conventional care only. A disappointing but important finding. However, by per-protocol analysis, the addition of OMT or of light touch decreased length of hospital stay, the duration of intravenous antibiotics and the incidence of respiratory failure and death relative to conventional care only. Further study is called for to explain these surprising results.

Meeting the need for randomized clinical trials of the role and efficacy of OMT is a responsibility of high priority for the osteopathic profession in this age of evidence-based medicine. The American Osteopathic Association (AOA) needs to consider reinstating a dues-generated financial set-aside both to increase its support of osteopathic research and to initiate a program of physician-investigator career development awards to recruit and help establish osteopathic clinical investigators in a career in translational and clinical research.

## Introduction

The publication of the paper by Noll et al [1] on the efficacy of osteopathic manipulative treatment (OMT) in patients with acute infectious pneumonia in *Osteopathic Medicine and Primary Care *raises two important issues: the results of the research *per se *and meeting the need for implementing additional clinical studies of osteopathic principles and practices.

## Results of a randomized controlled clinical trial

The pneumonias due to infection continue to be a meaningful threat to the health and viability of persons, particularly those in high risk groups: children, the aged and the debilitated. Osteopathic manipulative treatment has been used by osteopathic physicians in the past as a primary intervention and continues to be used at the present by them --- usually as an adjunct to antibiotic therapy. What is the evidence supporting the use of OMT as an effective clinical intervention?

Pneumonia is a generic term identified with an inflammation of the lung parenchyma characterized by consolidation of the affected part, the alveolar air spaces being filled with exudates, inflammatory cells, and fibrin. Most cases are due to infection by bacteria (e.g. *Streptococcus pneumoniae*) or by viruses; a few are due to inhalation of chemicals (e.g. chlorine), trauma to the chest wall, and a small minority to rickettsias, fungi and yeasts. In addition there are a number of other events that come under the broad heading of pneumonia such as the aspiration pneumonias, post-embolic pulmonary infarction and eosinophilic pneumonia. Diagnosis is dependent on the history of events leading up to the illness, on other pathologies preceding or present at the time of occurrence and on characteristic findings which can include: systemic symptoms of acute infection, a cough, dyspnea, auscultatory evidence of airspace filling and/or lobar consolidation, hypoxemia, and presence of an infiltrate on imaging of the chest. Thus, pneumonia describes a syndrome of several potential etiologies and pathogeneses. Conventional therapy includes eliminating the etiologic agent (e.g. antibiotic therapy for the bacterial pneumonias); also, the judicious use of supportive therapy (e.g. oxygen inhalation; parenteral fluids). In addition, vaccines are available for prevention of infectious pneumonias in high-risk populations. Other classes of interventions used in conjunction with conventional therapy are usually included under the classification "complementary medicine"; at this time, OMT is included in this classification.

Prior to the development and availability of the sulfa drugs in the 1930-1940s and the antibiotics in the 1950s, the infectious pneumonias were treated symptomatically addressing primarily the respiratory distress, fever and malaise usually associated with them. Herbs, chest counter-irritants (e.g. mustard, cupping), and therapeutic baths were commonly used therapeutic modalities as were a broad spectrum of naturopathic and homeopathic modalities. Death was not uncommon, particularly in children and the aged. It was in this era that osteopathic treatment was reported to be of significance in preventing death and assisting in recovery. Utilizing osteopathic principles of mobilizing total body resources, particularly neurological and immune system mobilization, OMT was administered regularly to the patient, emphasizing spinal mobilization in the thoracic area and manipulative procedures directed at increasing lymphatic flow. Positive results following OMT were reported in observational studies, including case reports and on occasion, case series. During this period, the principles of the controlled clinical trial had not yet been fully developed as a basis for evidence-based medicine and thus results from observational studies were the source of most clinical therapies --- including the use of osteopathic principles and OMT in the treatment of pneumonia.

The latter third of the 20^th ^century and the early part of the 21^st ^century are characterized in medicine by the application of the methodologies of the controlled clinical trial as the source of information for evidence-based medicine. During this period, the randomized, blinded, controlled clinical evaluation with sufficient power to meet statistical analysis needs has become the established standard for evaluation of a diagnostic, preventive or therapeutic modality. Variations from this standard were and are being developed to meet the needs of various situations in which the randomized controlled clinical trial would be difficult to utilize; examples are: rare diseases; disorders with a variety of characteristics as in some behavioral dysfunctions; diseases with a fixed end-point such as death or an anatomical structural defect. With full recognition of the valuable information accumulated over the years from observational studies, the randomized clinical trial has now become the standard for evidence-based medicine, including osteopathic clinical care. Both already established clinical interventions and newly developed potential interventions are now subject to evaluation by this research methodology.

Over the past two decades, a limited number of controlled clinical trials of manipulative treatment --- osteopathic, chiropractic and allopathic --- have been completed. Most trials addressed the issue of pain: headache, cervical pain, lower back pain; a few addressed systemic disease. However, despite this effort many suffered from a scarcity of the resources essential to the organization and conduct of controlled clinical trials: research design expertise; a sufficient number of patients; operational experience; statistical support; adequate funding. To meet these needs, the osteopathic profession has developed several national centers which can assist in mobilizing the resources necessary for the conduct of well-designed clinical trials to evaluate the efficacy of osteopathic diagnostic and therapeutic methodologies. The A.T. Still Research Institute at the Kirksville College of Osteopathic Medicine and The Osteopathic Research Center at the University of North Texas Health Science Center are examples of these. Both provide investigators with the technical assistance and support necessary to develop and conduct institutional and multi-institutional controlled clinical trials of osteopathic clinical care.

With participation of the A.T. Still Research Institute and assistance from The Osteopathic Research Center, a multi-institutional, blinded controlled clinical trial has been reported in 2010 to evaluate the efficacy of OMT in the treatment of pneumonia: "Efficacy of osteopathic manipulation as an adjunctive treatment for hospitalized patients with pneumonia: a randomized controlled trial." The participants were Donald R Noll, Brian F Degenhardt, Thomas F Morley, Francis X Blais, Kari A Hortos, Kendi Hensel, Jane C Johnson, David J Pasta and Scott T Stoll. The study included over 400 patients; 50 years of age or older; randomized to three intervention protocols: conventional care only (CCO), CCO plus OMT; and CCO plus light touch (LT). The data obtained indicate that by intention-to-treat (ITT) analysis, the addition of OMT to conventional care did not improve the designated outcomes when compared to conventional care only. By per- protocol (PP) analysis, the addition of OMT decreased length of hospital stay, duration of intravenous antibiotics and the incidence of respiratory failure and death relative to conventional care only. Thus, if a subject received the OMT plus conventional care protocol as prescribed without missing any treatment sessions, there was significant benefit; however, the same positive result was found with light touch. Why no difference in outcome between OMT and light touch, that remains to be studied. Was it because there are no differences, or because it is a result of research design? These are questions that now require a response.

## Evidence-based medicine: the evaluation of osteopathic principles and practice

The authors are to be congratulated for mastering the technology and the art of the randomized controlled trial (RCT) and for utilizing it for the evaluation of OMT in the treatment of a life-threatening acute illness. Modern medicine now requires that OMT's present and future clinical application be evaluated utilizing methodologies that are reliable and reproducible. The RCT is a critical methodology for meeting that objective. It is a methodology that the osteopathic profession must foster so that its identifying characteristics of patient care become part of conventional medicine rather than continuing to be considered complementary medicine.

The basic principles of the RCT are well established, although its application to a specific question requires a variety of specific expertise. However, the conduct of a RCT is an art form requiring the continuing interaction of a variety of committees to monitor the conduct of the protocol and to address the several operational problems that will undoubtedly arise during the conduct of the trial. The RCT is also usually very expensive and often dependent upon government funding. In order to be successful in its design, operation and funding, funds in support of a pilot study become an essential requirement. Once the proposed study --- its concept, objectives and potential have been tested, the probability of receiving adequate funding for the full study becomes more realistic. Meeting the need for pilot studies of RCT of OMT is a responsibility of the osteopathic profession --- including national, state and local osteopathic associations and the several private foundations associated with the profession.

The American Osteopathic Association (AOA) and its affiliates continue to provide funds for this purpose. However, the funds available are in short supply. In years past, the AOA established a special financial set-aside for osteopathic research by initiating a modest assessment attached to AOA dues. This assessment was time limited and has been discontinued. Should this mechanism for enlarging the resources available be reinstated by the AOA in order to stimulate additional osteopathic research? Should these funds be used also to establish a program of career development awards to prepare a cadre of osteopathic physician-investigators for future leadership in OMT research? I suggest it should. How else can the osteopathic profession meet its responsibilities to be active participants in what is rapidly becoming the basis for modern clinical care --- evidence-based medicine. The osteopathic profession through its national organization, the AOA, needs to move ahead aggressively to provide the resources necessary --- people and money --- for accelerating osteopathic participation in modern medical research, specifically the role and efficacy of OMT in the promotion of health and the treatment of illness.

## Competing interests

The author declares that they have no competing interests.

## Appendix

The author recommends the following general sources:

- Galen B.T. Approach to the patient with suspected pneumonia. In Kelley's Textbook of Internal Medicine, 4 ed. Lippincott Williams & Williams; 2000:2412-2419

- Mandell L.A. and Wunderink, R. Pneumonia. In Harrison's Principles of Internal Medicine, 7 ed. McGraw-Hill Companies; 2008:1619-1628

- Northup G.W. Osteopathic Medicine: An American Reformation (1970) American Osteopathic Association

- Rumney I.C. Osteopathic manipulative treatment of infectious diseases. In Stark E.H. and Tilley R.M. Clinical Review Series: Osteopathic Medicine. Insight Publishing Company; 1975:165-169
